# Adipose-derived mesenchymal stem cells and retinal pigment epithelial cells interactions in a stress environment via tunneling nanotubes

**DOI:** 10.1371/journal.pone.0329672

**Published:** 2025-08-04

**Authors:** Merve Gozel, Karya Senkoylu, Cem Kesim, Murat Hasanreisoglu

**Affiliations:** 1 Koç University Research Center for Translational Medicine, Koç University, Istanbul, Türkiye; 2 Koç University School of Medicine, Istanbul, Türkiye; 3 Department of Ophthalmology, Koç University School of Medicine, Istanbul, Türkiye; King Abdulaziz University, EGYPT

## Abstract

This study aims to demonstrate the formation of tunneling tubes (TNTs) between adipose-derived mesenchymal stem cells (AdMSCs) and retinal pigment epithelial cells (RPE-1) and their alterations in response to experimental stress conditions. Serum starvation was employed as a stress condition to induce TNTs between the AdMSC and RPE-1 cells. The presence of TNTs was demonstrated through immunofluorescence microscopy, while scanning electron microscopy was utilized to determine the average thickness. Cell viability was assessed after stress by CellTiter-Glo, and H2DCFH-DA probes evaluated the cells’ reactive oxygen species (ROS) levels. Further, JC-1 labelled mitochondrial exchange between cells via TNTs was confirmed by time-lapse imaging. A transmembrane culture system was employed to inhibit TNTs. In this study, we investigated the role of TNTs in facilitating intercellular communication and mitochondrial transfer between AdMSCs and RPE-1 cells under stress. We found that TNT-mediated mitochondrial transfer from AdMSCs to RPE-1 helps to reduce ROS levels and improve cell viability. We demonstrated that direct interaction between AdMSCs and RPE-1 cells was crucial for stress recovery. Co-culture enhanced the viability and sustained the RPE-1 cells’ function after stress-induced damage. Mechanical inhibition of TNT formation decreased cell viability and elevated ROS levels, indicating the importance of TNTs in cellular protection. The findings can provide a new perspective on the therapeutic potential of stem cell-based therapy in protecting retinal pigment epithelium cells against stress-induced damage and promoting tissue regeneration.

## Introduction

TNTs (tunneling nanotubes) are long cytoplasmic bridges that have a remarkable ability to conduct a wide range of functions that mediate cellular physiology and cell survival [1]. TNT appears to play a role in intercellular exchanges of signals, chemicals, organelles, and pathogens, involving them in a wide range of functions, according to a growing body of research [2]. They are crucial in embryonic development, cell migration, cell healing, cancer therapy resistance, and pathogen dissemination as intercellular bridges [2]. The presence of TNTs are confirmed in various ocular cells, including corneal [3], trabecular [4], and retinal [5] cells. The retinal pigment epithelium (RPE) is a monolayer of cells located at the posterior of the eye, between the retina and choroid, which has critical roles in vision [6]. Damage to the structure and function of the retinal pigment epithelium leads to a variety of retinal diseases, such as diabetic retinopathy (DR), age-related macular degeneration (AMD), or retinitis pigmentosa (RP). Its dysfunction is a key contributor to the onset and progression of multiple retinal degenerative diseases. In AMD, RPE atrophy disrupts photoreceptor maintenance, leading to central vision loss due to the degeneration of photoreceptors and choriocapillaris [7]. In RP, although the primary defect is in photoreceptors, secondary RPE degeneration accelerates disease progression and exacerbates visual decline [6]. In DR, chronic hyperglycemia induces oxidative stress and inflammatory responses in the RPE, compromising the outer blood–retinal barrier and contributing to vascular leakage, macular edema, and subsequent visual impairment [8]. These observations underline the importance of maintaining or restoring RPE integrity when developing therapeutic strategies for retinal diseases. Novel treatment strategies for restoring the structural and functional integrity of RPE cells lead to the ocular application of mesenchymal stem cells (MSCs). MSCs have been shown to have beneficial effects on damaged RPE cells. These effects include the differentiation of MSCs into RPE cells [9] and the regulation of the retinal medium through MSC-derived conditioned media [10]. Adipose-derived MSCs (AdMSCs) have been previously shown to establish TNTs directed to corneal epithelial cells to perform mitochondrial transfer, aiming for protection against mitochondrial damage [11]. AdMSCs are particularly advantageous due to their easy accessibility, high yield, and fewer ethical concerns compared to other stem cell sources [12]. Given that various cell types of the ocular tissue including RPE cells show the presence of TNT structures, and there is increasing evidence for MSCs to have TNTs as another mechanism of action aimed for cell and tissue regeneration, the question arises whether a similar mechanism will also be employed in retina pigment epithelium repair following retinal injury.

In this study, we aimed to determine whether AdMSC-mediated mitochondrial transfer could rescue the retinal pigment epithelium from several stress-induced mitochondrial damages. We established that AdMSCs could efficiently donate functional mitochondria and protect RPE-1 from serum starvation stress-induced damage through TNT formation.

## Materials and methods

### Ethics statements

This study adhered to national regulations and was approved by the Ethics Committee of the Koc University School of Medicine, Istanbul, Turkey (protocol no: 2020.447.IRB2.121, date of approval: 03.12.2020). Study subjects (8 adipose tissues from liposuction surgery) were recruited from Koç University Hospital, Plastic, Reconstructive and Aesthetic Surgery Department. The recruitment period for this study started on Jan 25, 2021 and ended on Nov 21, 2022 (The recruitment process was interrupted due to the COVID-19 pandemic). Written informed consent was obtained from all patients after an explanation of the nature and possible consequences of the study.

### Isolation and culture of adipose-derived mesenchymal stem cells

Adipose-derived mesenchymal stem cells (AdMSCs) were isolated, cultured, and characterized as previously described by our group [13]. Briefly, AdMSCs were isolated from liposuction samples obtained from healthy male and female donors (aged 20–50) and processed using collagenase digestion. Cells were cultured in Dulbecco’s Modified Eagle Medium – low glucose (DMEM-LG; Biowest, Nuaille, France) supplemented with 10% (v/v) Fetal Bovine Serum (FBS; Biowest, Nuaille, France) and passaged until P4 for co-culture experiments. Characterization of AdMSCs was performed via flow cytometry using positive markers (CD73, CD90, CD146) and negative markers for hematopoietic cells (CD3/4/8, CD56, CD20). Differentiation potential into osteogenic, chondrogenic, and adipogenic lineages was assessed using commercial differentiation kits, with lineage-specific markers (Alizarin Red S, Alcan Blue, and Oil Red O) confirming differentiation through microscopy imaging.

### Culturing of retinal pigment epithelial cell line (RPE-1)

RPE-1 cells (ATCC® CRL‐4000) were grown in DMEM/F12 (Dulbecco’s MEM; PAN Biotech, Aidenbach, Germany) supplemented with 10% fetal bovine serum (Biochrom, Berlin, Germany), 100 IU/ ml penicillin, 100 μg/ml streptomycin, and were used at passages 10–15. The viability of cultured cells was assessed microscopically by the trypan blue exclusion test.

### In vitro co-culture systems

#### Oxidative stress model and in vitro co-culture system.

RPE-1 cells with (oxidative stress) or without (normal condition) hypoxia treatments were co-cultured with AdMSCs at a 1:1 ratio. AdMSCs were used in passage 4. RPE-1 cells were also cultured alone for control. The mixed cells (co-cultured cells) were seeded at a density of 2 × 10^5^/cm^2^ per well in a plate and cultured with an AdMSC medium supplemented with DMEM-LG with 10% FBS, 100 IU/ ml penicillin, 100 μg/ml streptomycin. The cells were incubated in a cell culture incubator containing 1% O_2_ for 48 hours at 37 °C.

#### Serum starvation model and in vitro co-culture system.

RPE-1 cells with or without a serum-free medium (serum starvation, SF) were co-cultured with AdMSCs at a 1:1 ratio. The mixed cells were seeded at a density of 2 × 10^5^/cm^2^ and cultured with a serum-free medium supplemented with DMEM-LG with 100 IU/ ml penicillin and 100 μg/ml streptomycin. RPE-1 cells were also cultured alone. The cells were incubated in a cell culture incubator for 48 hours at 37 °C.

### Cell viability assay

CellTiter-Glo assay (CTG, Promega, G7570) was performed for cell viability. CTG reagent was mixed at a 1:10 ratio with supernatant from the treatment plate. The mix was incubated for 2 min at room temperature on the shaker and then allowed the plate to incubate at room temperature for 10 min to stabilize the luminescent signal, followed by luminescence measurement.

### Evaluation of reactive oxygen species (ROS)

RPE-1 cells with or without AdMSCs were seeded in 96-well plates and exposed to stress conditions for 48 h. The cells were then incubated at 37 °C and 5% CO_2_ for 30 min with the 2,7-dichlorodihydrofluorescein diacetate (H_2_DHF-DA) probe. At the end of the incubation, the medium was removed and replaced with the new medium. The fluorescence intensity was measured through a fluorescent Synergy-H1 Microplate Reader (Bio-Tek Instruments) with an excitation wavelength of 485 nm and an emission wavelength of 538 nm.

### Microscopic analysis

#### Immunofluorescence (IF) staining.

Co-cultured cells and control groups were fixed with 4% paraformaldehyde (PFA) for 20 min at room temperature. Then, permeabilization of the cells was performed with 1% Triton X-100 (Sigma-Aldrich, T8787) in 1x DPBS for 7 min, and nonspecific antibody binding was blocked by incubating samples with a superblock solution (Scytek, AAA125) for 10 min. Then, cells were stained with primary antibodies and incubated for 90 min at 37 °C in a 1:50 dilution. To visualize the actin cytoskeleton, cells were incubated with monoclonal mouse anti-β-tubulin antibody (sigma, T9026), and negative control reactions were incubated with phosphate-buffered saline (PBS) instead of the primary antibody. The secondary antibody, anti-mouse (1:100), was then used against the primary antibodies. Texas Red™-X Phalloidin (ThermoFisher, T7471) was used for binding F-actin of tunneling nanotubes and DAPI for nuclei.

#### Quantification of TNTs.

The numbers of TNTs in the IF-labelled populations were counted and expressed as the number of TNTs per 100 cells. Co-cultures of AdMSC and RPE-1 cells were incubated under stress. For counting of TNTs, AdMSC and RPE-1 cells were co-cultured for 48 h in the presence of serum-free media. Afterward, cells were stained with anti-β-tubulin and Texas Red™-X Phalloidin and incubated for 90 min at 37 °C, followed by immunofluorescence imaging using the Leica TCSSP5 confocal microscope (TCS SP8 DLS, Leica) equipped. 20 images containing at least 350 cells were obtained in each condition.

#### Scanning electron microscopy (SEM) imaging.

The media was discarded, and the cells were washed with Dulbecco’s Phosphate-Buffered Saline (DPBS). 2% PFA was prepared from 16% PFA solution (Paraformaldehyde 16% Solution, EM Grade, Electron Microscopy Sciences, Hatfield, PA) in nanopure water. The cells were fixed using a 2% PFA solution and let air dry for 2 d. SEM imaging was performed by a ZEISS EVO LS15 scanning electron microscope.

#### Assessment of mitochondrial transfer.

Briefly [5], for live time-lapse imaging, co-cultured cells were monitored with a confocal microscope (TCS SP8 DLS, Leica) equipped with Differential Interference Contrast (DIC) components. Cells were imaged every 4 min for 30 min at 37 °C and 5% CO_2_. Image analysis was performed using ImageJ/Fiji software (Version 2.0 [14]). To stain mitochondria, cells were labelled with 1 mM JC-1 (Invitrogen) for 15 min at 37°C and visualized with a confocal microscope. JC-1 selectively accumulates within the mitochondrial matrix and forms red fluorescent J-aggregates (emission 590 nm) in the presence of negative transmembrane potential but exists as green monomers (emission 530 nm) under depolarised conditions. The green phase was recorded using an excitation wavelength of 485 nm and an emission filter of 540/550 nm. The red phase of JC-1 was recorded using an excitation wavelength of 535 nm and an emission filter of 610/675 nm.

### Transmembrane (Transwell^®^) culture assay

Cell-cell contact (TNT formation) is prevented by using two types of insert models with a 0.4-μm pore polyester membrane (Transwell^®^, SARSTEDT) in a 24-well plate. AdMSCs were cultured on the upper surface of each insert membrane, and RPE-1 cells were seeded on the surface of a 24-well plate (Fig 5A), and vice versa (Fig 5B). In the first set-up, AdMSCs/RPE-1 were allowed to co-culture for approximately 48 h, followed by removal of the insert, and RPE-1 cells were analyzed for cell viability and ROS detection assays as described above.

**Fig 1:**
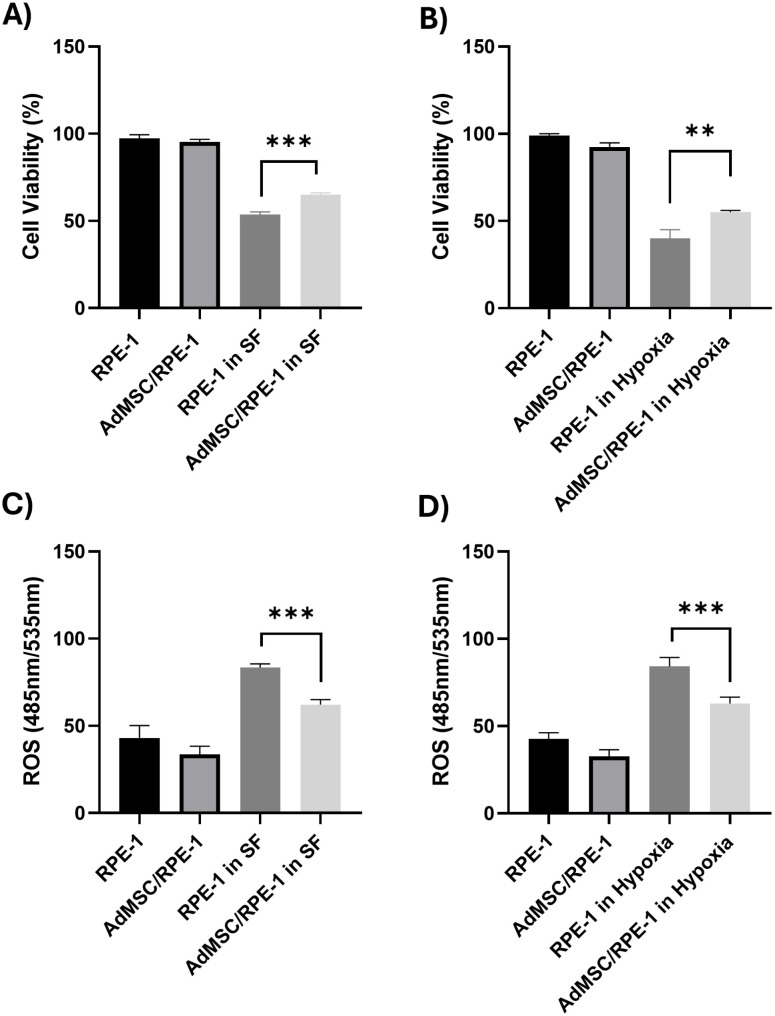
Cell viability and comparison of cells’ reactive oxygen species (ROS) in stress environments. (A-B) Retina pigment epithelium-1 (RPE-1: monoculture) and co-cultured (AdMSC/RPE-1) cells were treated with serum-free (SF) and hypoxic (hypoxia) media for 48h. Cell viability assay was measured by CellTiter-Glo (CTG) at 48 hours. The percentage of cell viability in comparison to the RPE-1 in their basal media is presented. Data are presented as mean ± standard deviation (S.D). Analysis of significance was performed by one-way ANOVA with Tukey’s multiple comparison test. Results are presented as ** P < 0.01 and *** P < 0.001. Cell viability was significantly increased under stress conditions in the co-cultured cells. (C-D) The change of ROS levels after 48 h of incubation in stress environments were determined by H_2_DHF-DA assay. The percentage of ROS in comparison to the basal ROS of RPE-1 in conditioned media is presented. Data are presented as mean ± standard deviation (S.D). Analysis of significance was performed by one-way ANOVA with Tukey’s multiple comparison test. Results are presented as *** P < 0.001. ROS levels were significantly decreased under stress conditions in the co-cultured cells.

**Fig 2:**
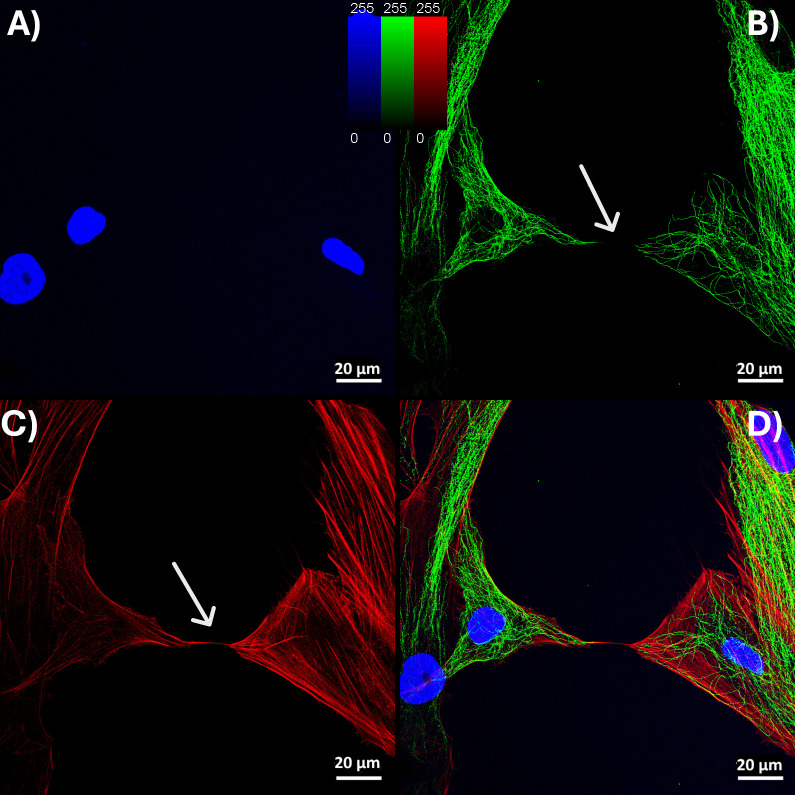
Tunneling Nanotubes (TNTs) form between the AdMSC and RPE-1 cells. Immunofluorescence (IF) images of AdMSCs and RPE-1 cells stained with (A) DAPI (blue) for nuclei, (B) anti-β-tubulin (green) for tubular structures, and (C) Texas Red™-X Phalloidin (red) for F-(actin). (B) IF images of two cells connected with TNT revealed the lack of β-tubulin in the TNTs (arrows) and (C) the presence of F-(actin) fibres within TNTs (arrows). (D) Merged pictures with F- (actin) (red), ß-tubulin (green), and nucleus (blue). Scale bars: 20 μm.

**Fig 3:**
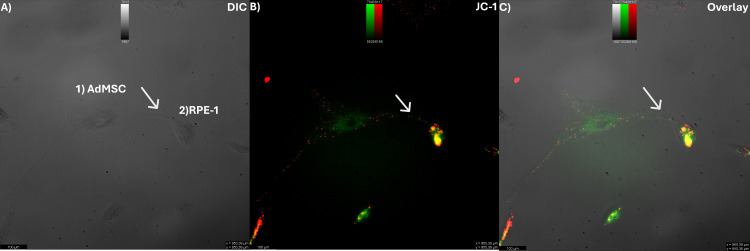
AdMSCs and RPE-1 are connected by tunneling nanotubes (TNT) containing mitochondria in serum starvation. (A) The bright field image shows two cells (1; AdMSC, 2; RPE-1) connected by a tunneling nanotube (arrow). (B) The corresponding fluorescence image shows JC-1 labelled mitochondria of cells (arrow). (C) The overlay of (A) and (B) shows the co-localization of nanotubes with fluorescent-labelled mitochondria. Scale bar: 100 µm.

**Fig 4:**
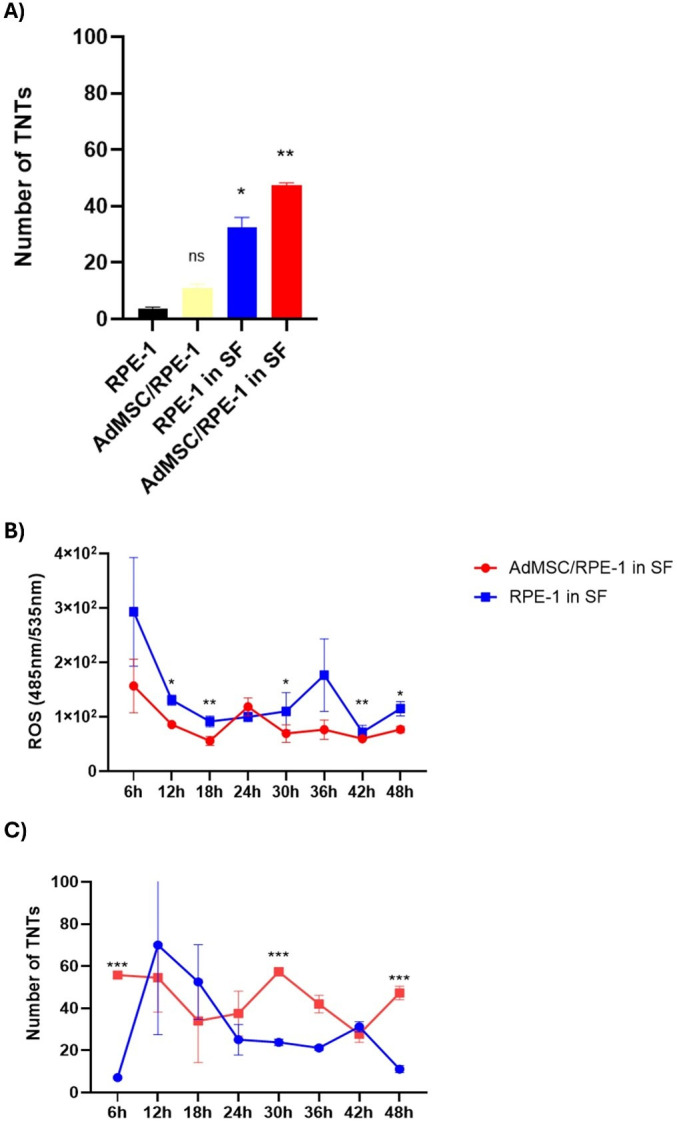
Effect of quantification of TNTs and their relationship with ROS levels. TNT structures were counted by immunofluorescence assay to show whether the survival of cells was related to TNTs. ROS levels were determined by H_2_DHF-DA assay. (A) TNTs were generated more in the co-cultured cells. Data are presented as mean ± standard deviation (S.D). For comparisons between co-cultured and RPE-1 cells in serum starvation, one-way ANOVA with Dunnett’s test was applied. All groups were compared to the RPE-1 cells in their basal media. ns P > 0.05, * P < 0.05, ** P < 0.01. (B) Time-dependent reactive oxygen species (ROS) levels and (C) the number of TNTs were evaluated under serum starvation. Time and its interaction on the measured variable, a two-way analysis of variance (2-way ANOVA) was conducted. Post hoc multiple comparisons were performed using Sidak’s correction to identify significant differences between groups at each time point. Data are presented as mean ± standard deviation (S.D.). Results are presented as * P < 0.05, ** P < 0.01, ***P < 0.001.

### Statistical analysis

Statistical analysis was performed using the Prism 8.02 (263) Software (GraphPad Software for Windows, San Diego, CA, USA). Data were presented as mean ± standard deviation (S.D). Mean values were calculated from at least three independent experiments (n ≥ 3), unless stated otherwise. Outliers were identified using Grubb’s Test at a significance level of α = 0.05. Normal distribution of data was demonstrated via the Shapiro-Wilk Test. In all cases, the null hypothesis that the data population is normally distributed was not rejected (p > α = 0.05). Analysis of significance was performed by one-way ANOVA with Tukey’s multiple comparison test or Dunnett’s test for comparisons involving more than two groups. For comparisons between two groups, either Student’s *t*-test or two-way ANOVA was applied, depending on the experimental design. A *p*-value < 0.05 was considered statistically significant. Levels of significance were defined as follows: * P < 0.05, ** P < 0.01, and *** P < 0.001.

## Results

### Cellular viability of RPE-1 cells in stress environments

Tunneling nanotubes were induced by serum starvation and oxidative stress. Cell survival was determined by CTG assay. The cell viability of RPE-1 cells seeded under stress without AdMSCs (indicated as RPE-1) was lower than that of the co-cultured (AdMSC/RPE-1) (Fig 1A,B).

### Reactive oxygen species (ROS) levels in the co-culture group

Elevated intracellular ROS level causes macromolecule disruption, cell cycle arrest, and DNA damage, leading to the selective death of cells [15]. 2,7-dichlorodihydrofluorescein diacetate (H_2_DCFH-DA) was employed to investigate the generation of ROS. In the presence of ROS, the H_2_DCF-DA molecule was converted to fluorescent dichlorofluorescein (DCF). The DCF fluorescence intensity was measured. Total ROS production in RPE-1 under stress conditions was significantly lower in the AdMSC/RPE-1 compared to RPE-1 (Fig 1C,D).

### Formation of TNTs between the AdMSC and RPE-1 cells

The immunofluorescence (IF) technique identified TNTs between the RPE-1 cells and AdMSCs. Co-cultured cells were found to be frequently interconnected by TNTs. Fluorescence microscopy revealed that TNTs, which are not anchored to the substratum, regularly connect cultured RPE-1 and AdMSCs in stress environments and contain F-actin but no microtubules (Fig 2B,C). Scanning electron microscope (SEM) showed the ultrastructure of TNTs as straight connections between cells with a diameter ranging from 30 to 300 nm and a length of up to 120 mm (S1 Fig).

### Mitochondrial transfer from AdMSCs to RPE-1

The change in ROS levels in co-cultured cells suggests that the observed effects could be attributed to the transfer of mitochondria between the cells or the production of antioxidants by MSCs [16], for which we addressed TNTs formation between AdMSCs and RPE-1 cells in co-culture. By using the specific mitochondrial dye JC-1, we observed fluorescently labelled mitochondria inside TNTs of living cells and demonstrated that TNTs allowed mitochondria transfer from the AdMSCs to RPE-1 cells in serum starvation (Fig 3 and S1 Movie).

### TNT formation increased in co-cultured cells compared to RPE-1, and it contributed to lower levels of ROS

To reveal the importance of TNT formation on the cells, we quantitatively analysed the number of TNTs between AdMSCs and RPE-1 cells for 48 h. TNTs increased under serum starvation in both RPE-1 and co-cultured cells (AdMSC/RPE-1) (Fig 4A). We have compared the fluctuation in ROS levels and the number of TNTs to interpret them in a time-dependent manner. When exposed to the stressful environment, cells in the co-culture were found to be more stress-resistant than the RPE-1 monoculture. The stress response that started in the first 6h was reduced by 50% in the co-culture group at the end of 48 h, while it was reduced by 45% in RPE-1 monoculture (Fig 4B). Simultaneously, the TNTs had a more stable trend in the co-cultured cells. The initial number of co-cultured-TNTs was higher than the RPE-1 monoculture (Fig 4C).

### The Transwell^®^ culture system allowed TNT inhibition between the cells

**Fig 5:**
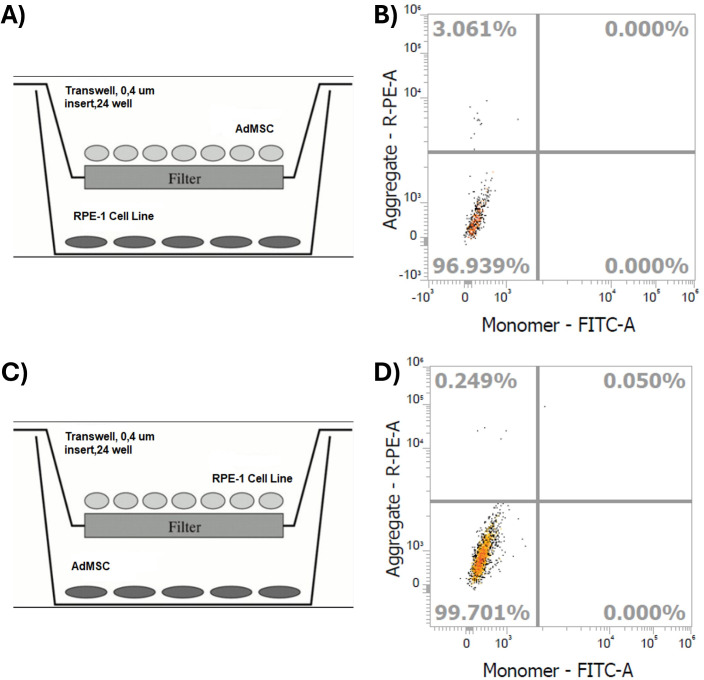
Schematic illustration of Transwell^®^ culture systems and effective inhibition of TNTs. (A, C) Transwell® membrane inserts (0.4 μm pore size) were used to physically separate the two cell types and to prevent direct cell-cell contact, thereby inhibiting tunneling nanotube (TNT) formation under serum starvation. In (A), AdMSCs were seeded in the insert and RPE-1 cells on the plate surface, whereas in (C), this design was reversed. (B, D) To evaluate mitochondrial transfer through TNTs, AdMSCs were labeled with JC-1 dye, which accumulates in mitochondria depending on their membrane potential. Following Transwell culture, the presence of JC-1-labeled mitochondria in RPE-1 cells was assessed by flow cytometry. Dot plots show fluorescence intensity for JC-1 aggregates (PE-A channel, indicating polarized mitochondria) and monomers (FITC-A channel, indicating depolarized mitochondria). Very low percentages of PE-positive RPE-1 cells (upper left quadrants; 0.029% and 0.249%) suggest there is no mitochondrial transfer, supporting the effective inhibition of TNTs under these Transwell culture system.

AdMSCs were stained with JC-1 and cultured with RPE-1 cells using the Transwell® system. Flow cytometry detected whether there is mitochondrial transfer from JC-1-stained AdMSCs to RPE-1 cells or not. If TNTs had formed, labelled mitochondria would be expected to transfer into RPE-1 cells, resulting in a detectable JC-1 signal (Fig 5B and 5D). However, since the Transwell® membrane prevents direct cell-to-cell contact and thereby inhibits TNT formation, no significant JC-1 signal was detected in RPE-1 cells. This absence of signal confirmed that mitochondrial transfer did not occur and that TNT formation was effectively prevented (Fig 5). Therefore, system (A) was chosen for further experiments, as it allowed direct access to and analysis of RPE-1 seeded on the plate.

To support the inhibition of TNTs via inserts, we performed time-dependent live-cell microscopy images showing mitochondrial dynamics in both co-culture and transwell culture systems of RPE-1 and ADMSCs. In the direct co-culture, JC-1-labeled mitochondria originating from ADMSCs were detected within RPE-1 cells over time, indicating successful mitochondrial transfer. However, such transfer was not observed in RPE-1 cells grown in the transwell culture system, supporting that physical separation prevented TNT formation and subsequent organelle exchange (S3 Fig).

### Transwell^®^ culture system failed to improve cell viability and suppress ROS levels

**Fig 6:**
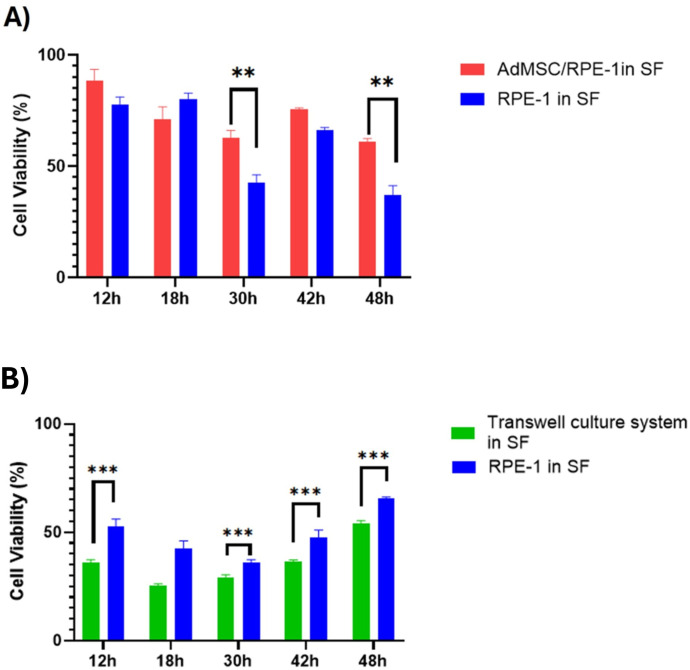
Comparison of the effects of co-cultured cells and Transwell^®^ culture system on RPE-1 viability. (A) RPE-1 cells were seeded either in direct co-culture or in a (B) Transwell® system with AdMSCs. Cell viability was measured in a time-dependent manner using the CellTiter-Glo (CTG) assay. Data are presented as mean ± standard deviation (S.D.). Statistical significance between the two groups at each time point was assessed using Student’s t-test. Results are presented as **P < 0.01, ***P < 0.001.

Based on the results shown in Fig 4B, cell viability and ROS levels of the RPE-1 of the Transwell^®^ culture system were analysed time-dependently by selecting the hours (12h, 18h, 30h, 42h, 48h) with statistically significant ROS levels. In serum starvation, RPE-1 cells exhibited higher viability when co-cultured with AdMSCs. However, the viability of RPE-1 significantly decreased when separated by the Transwell® insert, even compared to RPE-1 cultured alone (Fig 6A,B). This became statistically significant after 30h and was sustained throughout the later phases of the experiment.

**Fig 7:**
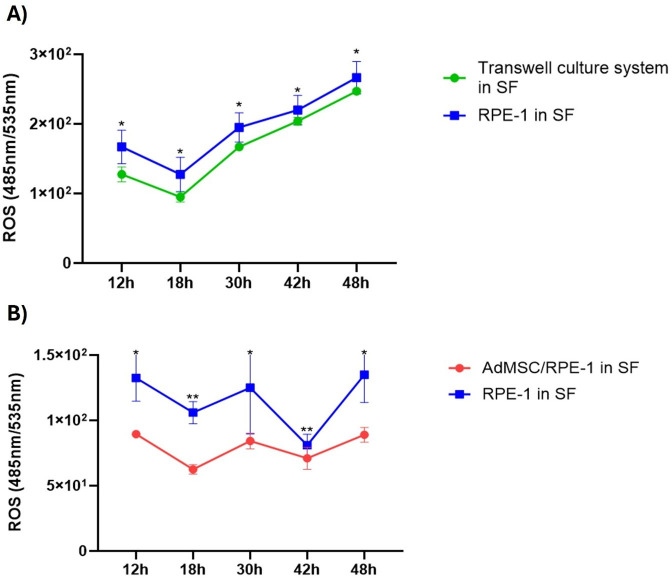
ROS changes of RPE-1 as time-dependently in the Transwell^®^ culture system and co-culture. ROS levels in RPE-1 cells under serum starvation were assessed over time in both Transwell® (A) and co-culture (B) conditions. Notably, ROS levels were not reduced when cells were physically separated by the Transwell® insert, in contrast to the co-cultured. Time and its interaction on the measured variable, a two-way analysis of variance (2-way ANOVA) was conducted. Post hoc multiple comparisons were performed using Sidak’s correction to identify significant differences between groups at each time point. Data are presented as mean ± standard deviation (S.D.). Results are presented as *P < 0.05, **P < 0.01.

To support the idea that AdMSCs need to contact RPE-1 to rescue retinal cells from stressful environments, we examined the time-dependent changes in ROS levels. Fig 7A showed that the ability of retinal cells alone to cope with stress was still harder than those recruited in the Transwell^®^ culture system. Nevertheless, the Transwell^®^ culture system had a limited effect to reduce the stress of the cells for the first 18h, which remained insufficient afterwards. On the other hand, direct communication of cells in co-culture managed to keep ROS reduced when compared to baseline (Fig 4B and 7B).

## Discussion

TNTs are crucial structures for efficient cell-to-cell communication, facilitating the exchange of signals, chemicals, organelles, and pathogens among adjacent or distant cells. In ocular tissues, including corneal, trabecular, and retinal cells, the presence of TNTs has been confirmed, emphasizing their significance in ocular physiology and pathology. Herein, we focused on the relationship of AdMSC-therapies with TNTs on RPE cells. The current study highlights that our co-culture experiments demonstrated that AdMSCs form TNTs with RPE-1 cells (Fig 2) and donate healthy mitochondria (Fig 3), leading to reduced ROS and improved RPE viability under stress (Fig 1). This is consistent with previous reports that RPE cells form TNTs capable of organelle exchange [5,17], and calcium signals among various cell types, including immune cells, cardiomyocytes, and epithelial cells [5,18,19]. Increased viability and a reduction in ROS are consistent with the idea that healthy mitochondria restore recipient cells’ oxidative phosphorylation and ATP synthesis [17]. Since impaired mitochondria are a significant source of ROS due to disrupted oxidative phosphorylation, replacing them with functional mitochondria can effectively reduce ROS production.

Afterward, we focused on examining the relationship between the quantity of TNTs and changes in ROS levels and cell viability. In the literature, increased nanotube numbers and reduced kidney tissue damage were observed in mouse kidney tissue under elevated oxidative stress [20]. Similarly, in our study, the number of TNTs increased in co-cultured cells after 48h serum starvation (Fig 4A). However, the ROS reduction did not scale linearly with the number of TNTs (Fig 4B,C), suggesting additional protective factors. For example, cytokine profiling showed that MSC co-culture downregulated pro-inflammatory mediators in RPE cells [21,22], reflecting an immunomodulatory effect. On the other hand, the cell survival of RPE-1 can also be associated with other organelles such as ribosomes, Golgi vesicles, and macromolecules, such as nucleic acids and proteins [23]. Overall, our findings support TNT-mediated mitochondrial transfer as a crucial mechanism of MSC-induced RPE rescue, but they also highlight cooperative paracrine and anti-inflammatory contributions. Mitochondrial transfer successfully survived damaged cells afflicted with dysfunctional mitochondria by restoring their mitochondrial function, demonstrated through enhanced oxidative phosphorylation and increased ATP generation [24]. Some studies showed that overexpression of calcium‐sensitive adaptor proteins enhances the transfer of mitochondria from MSCs to neuron‐like cells experiencing ischemic damage, improving recovery and cell proliferation [25,26]. Similarly, co-cultured mouse cardiomyocytes with AdMSC demonstrated that mitochondrial transfer from stem cells to cardiomyocytes plays a role in cardiomyocyte reprogramming [27]. By explaining intercellular mitochondrial trafficking from MSCs to corneal endothelial cells, photoreceptors, and retinal pigment cells, our study builds on earlier discoveries. According to recent studies, recipient cells that obtained mitochondria from MSCs demonstrate optimized respiratory capacities for the mitochondria and a greater expression of genes related to the structure and function of the mitochondria, suggesting the therapeutic potential of MSC-based mitochondrial transfer in the regeneration of ocular tissue [11,28].

The ability of AdMSC-derived mitochondria to empower recipient RPE cells to cope with increased ROS levels is a central aspect of our study. By enhancing cellular antioxidant defence mechanisms and maintaining redox balance, we demonstrated the capacity of AdMSC-mediated mitochondrial transfer to mitigate oxidative stress-induced RPE damage (Fig 4B, S2 Fig). Similarly, studies on CECs have shown that mitochondrial transfer from AdMSCs reduces ROS levels and preserves mitochondrial function under oxidative stress, highlighting the therapeutic potential of this approach in protecting RPE against mitochondrial damage [11].

Therefore, indicating that intercellular mitochondrial transport is a vital mechanism for the regeneration of corneal epithelial cells and retinal ganglion cells, further supporting the role of mitochondrial transfer in ocular tissue repair. While our study provides strong evidence that mitochondrial transfer via TNTs, a limitation is that the observed increase in ATP levels only suggests enhanced mitochondrial activity without directly measuring it. This indirect evidence does not substitute for direct assessments of mitochondrial function. Parameters such as oxygen consumption rate, extracellular acidification rate, or mitochondrial membrane potential would provide a more comprehensive understanding of the bioenergetic contributions of the transferred mitochondria. Future studies should include such assays to further elucidate the underlying mechanisms and therapeutic relevance of mitochondrial transfer.

Alternatively, during 48 hours of serum starvation, we observed the bidirectional mitochondrial exchange between AdMSCs and RPE-1 cells at various intervals (S2 Fig). In vitro investigations revealed that mitochondrial transport can be bidirectional, which was previously demonstrated by the transfer of mitochondria between renal tubular cells and mesenchymal multipotent stromal cells [29]. This exchange increased MSC proliferation [30] and suggests a mutually beneficial effect on both cell populations in stress.

To show the importance of TNTs, we forced a mechanical inhibition via the transwell culture system (Fig 5). The Transwell membrane filter can act as an efficient physical barrier to TNT formation [31]. After successfully inhibiting TNTs, RPE-1 cells’ viability (Fig 6) was decreased dramatically, and ROS levels were elevated (Fig 7) with the transwell culture system. Indeed, several investigations have demonstrated the relevance of direct cell-cell contact in enabling metabolite transfer between cells, which can improve cell survival and function [31]. Therefore, we presume that a direct connection between these cells is essential. Furthermore, in co-culture systems or multicellular aggregates, physical contact between cells has been found to improve cell survival and functional outcomes as compared to cultures in which cells are spatially separated [31]. This shows that direct cell-to-cell interactions in stem cell treatment which allow TNT-mediated intercellular transfer could have advantages against indirect application of stem cell-based products such as exosomes [32] for improving cellular responses to environmental stimuli and stresses in several tissue types as retinal pigment epithelium on the clinical applications. From the clinical perspective, it could be emphasized that the success of stem cell-based products on treatment of retinal diseases should also be dependent on the ability to form direct cell-to-cell connections between stem cells and target retinal cells according to different delivery methods including subretinal, intravitreal or suprachoroidal approaches [33]. Our findings highlight the importance of physical contacts between cells in facilitating intercellular communication and metabolic cooperation.

Conversely, in most of the studies, cell-free therapy seems to be favoured in regenerative medicine after the discovery that transplanted cells exert their therapeutic effects mainly through the secretion of paracrine factors [34]. Our study has co-cultured and transwell systems to specify the effects of soluble factors versus direct cell-cell interactions. The transwell system enables the evaluation of MSC-secreted soluble factors by preventing direct cell-to-cell contact, allowing us to specifically assess their individual contributions. Several studies have demonstrated the efficacy of MSC-conditioned media (CM) collected after shorter incubation periods (24 h) in promoting cell proliferation, migration, and immunomodulation [35,36]. For instance, researchers [37] reported that 24h MSC-CM enhanced the proliferation and migration of alveolar epithelial cells via the JNK-P38 signaling pathway. Similarly, other groups [38] showed that MSC-CM improved the functions of hyperglycemic fibroblasts, including proliferation and migration. However, our experimental design involved a 48h incubation period. Literature suggests that the biological activity of MSC-CM may diminish over extended incubation times, potentially due to the degradation of soluble factors or changes in their composition. This attenuation could explain the reduced efficacy observed in our 48h insert assay. Consequently, our findings indicate that while MSC-derived soluble factors contribute to cellular responses, direct cell-cell interactions become increasingly significant over prolonged incubation periods. This is particularly relevant in the context of tunneling nanotube formation, which facilitates direct intercellular communication and has been implicated in various physiological processes.

## Conclusion

Our study provides evidence that TNT-mediated mitochondrial transfer from AdMSCs to RPE-1 cells supports cellular stability under stress. While mitochondrial transfer appears to be a vital mechanism, complementary roles of soluble factors, calcium signaling, and bidirectional communication must also be considered. Future research should validate these findings in vivo and investigate therapeutic strategies leveraging TNTs for the targeted delivery of organelles and biomolecules in retinal degenerative diseases.

## Supporting information

S1 Fig The presence of TNTs in AdMSC and RPE-1 cells by scanning electron microscopy (SEM). (PDF)

S2 Fig Mitochondrial transfer from AdMSCs to RPE-1 cells has been shown in a time-dependent manner. (TIF)

S3 Fig Time-dependent live-cell microscopy images from the transwell culture system and the direct co-culture of RPE-1 and AdMSCs. (PDF)

S1 Movie Time-lapse video monitoring of mitochondrial dynamics between AdMSC and RPE-1 cells. (AVI)

S1 Dataset Minimal data set related to experiments on TNT-mediated interactions between AdMSCs and RPE-1 cells under stress conditions. (XLSX)
