# Supplementary figures and images for: Adipose-derived mesenchymal stem cells and retinal pigment epithelial cells interactions in a stress environment via tunneling nanotubes
Source: PLoS One. 2025 Aug 4;20(8):e0329672. doi: 10.1371/journal.pone.0329672 (PMC12321103; doi:10.1371/journal.pone.0329672)

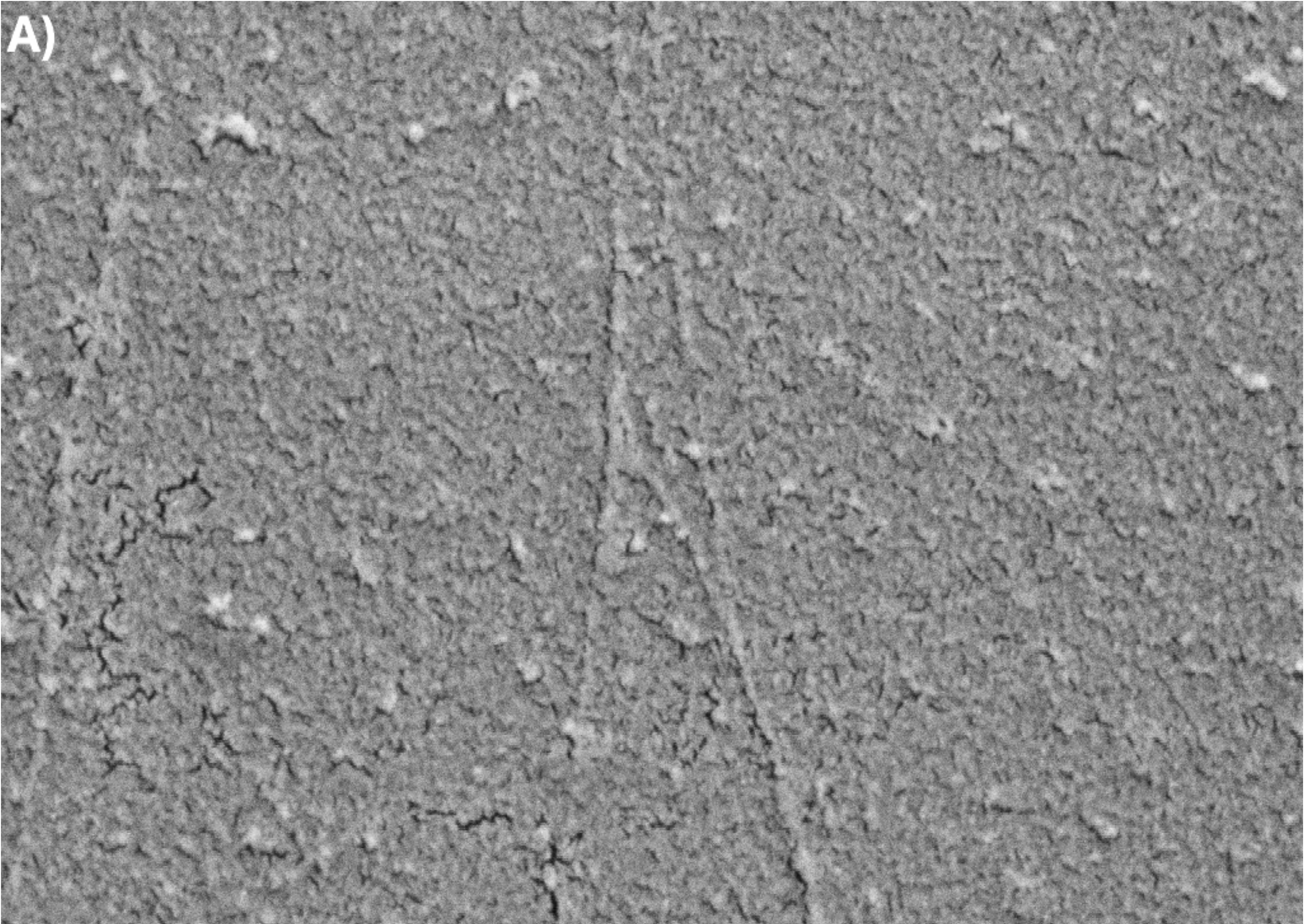

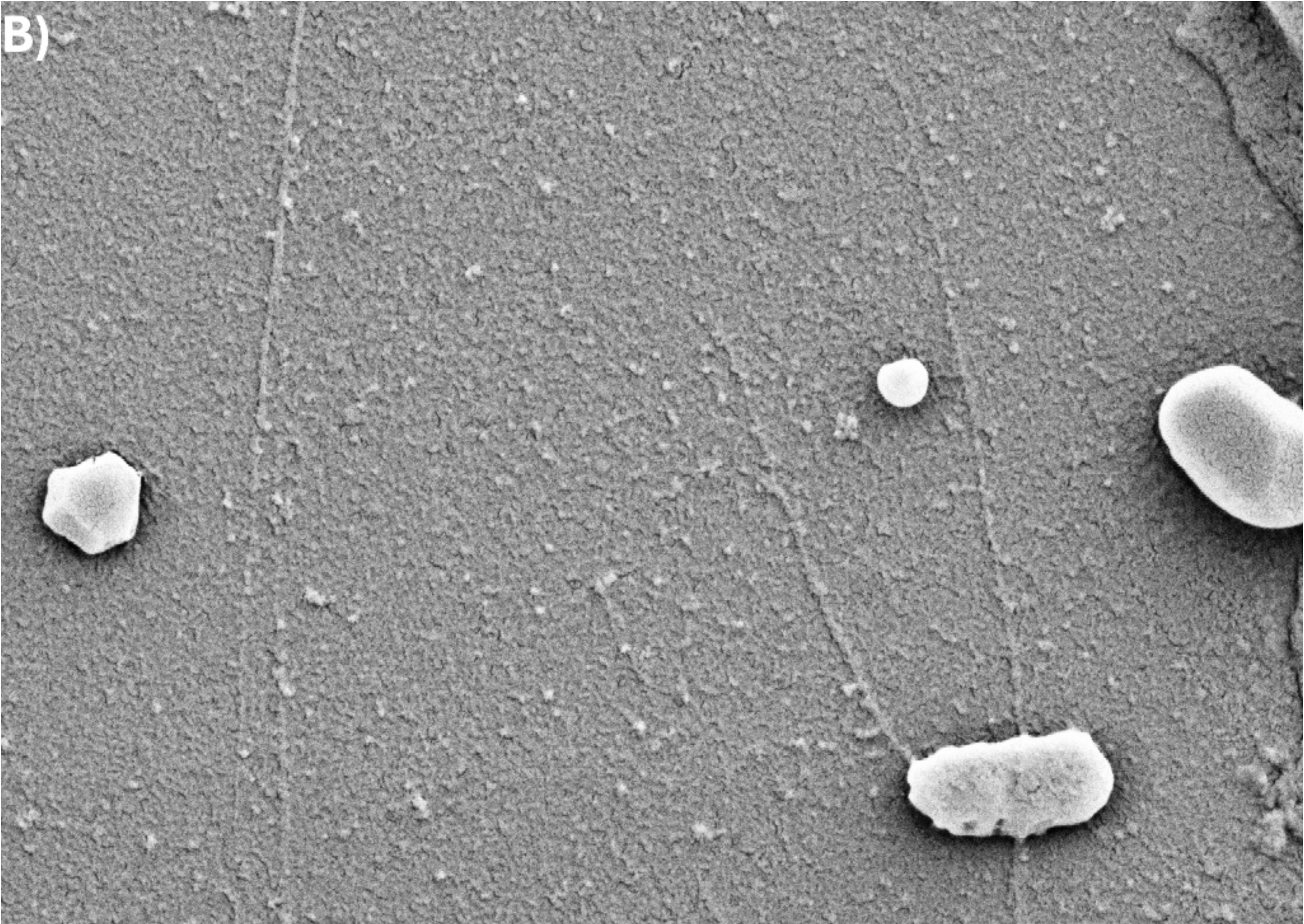

200 nm  
└───┘

50.00 K X

EHT = 3.00 kV

Signal A = SE2

WD = 6.5 mm

I Probe = 200.0 nA

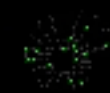 KUYTAM

Supplement: S1 Fig — (PDF) [file pone.0329672.s001.pdf]

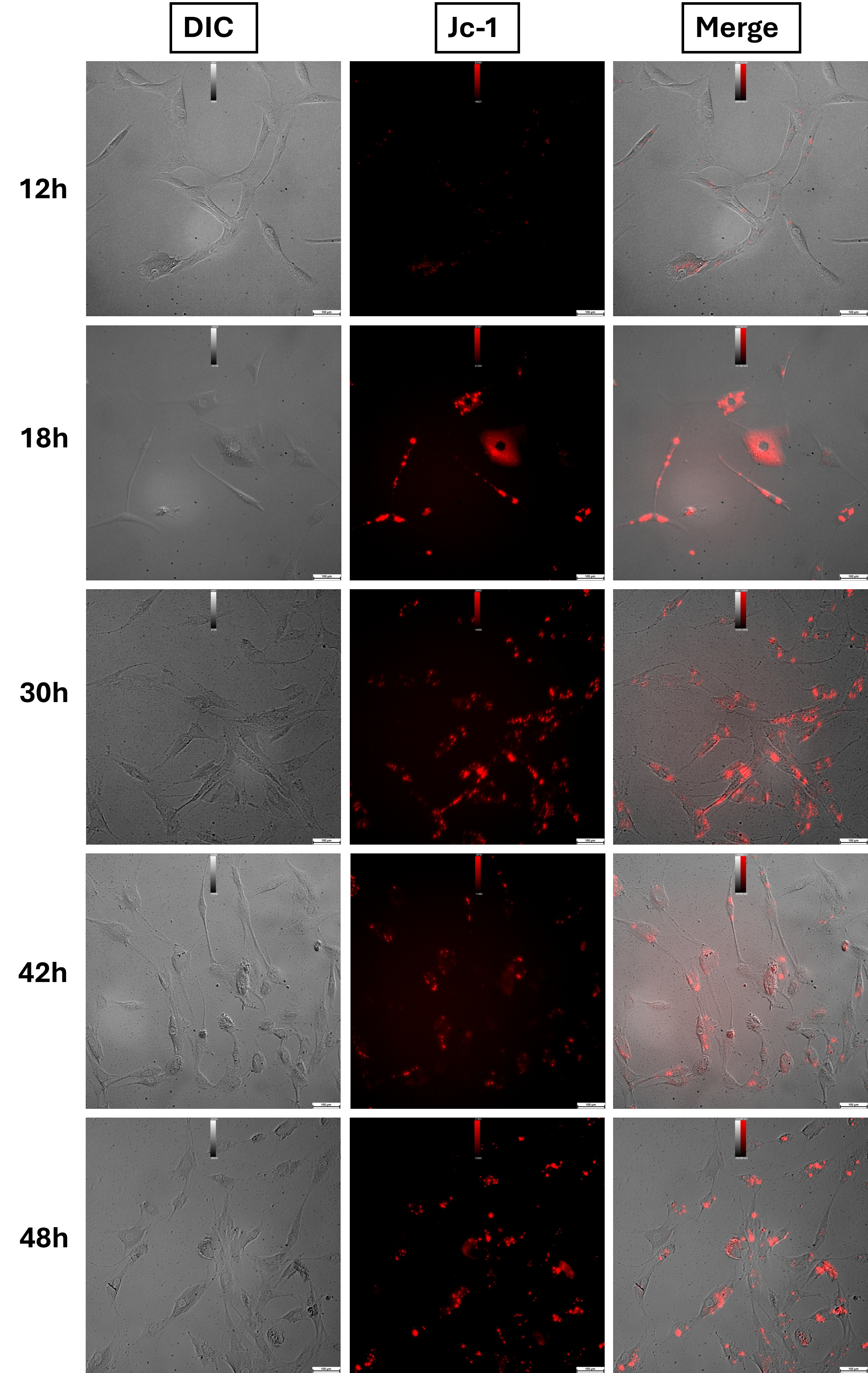

Supplement: S2 Fig — (TIF) [file pone.0329672.s002.tiff]
