# Supplementary material for: Adipose-derived mesenchymal stem cells and retinal pigment epithelial cells interactions in a stress environment via tunneling nanotubes
Source: PLoS One. 2025 Aug 4;20(8):e0329672. doi: 10.1371/journal.pone.0329672 (PMC12321103; doi:10.1371/journal.pone.0329672)
Supplement: S3 Fig — (PDF) [file pone.0329672.s003.pdf]

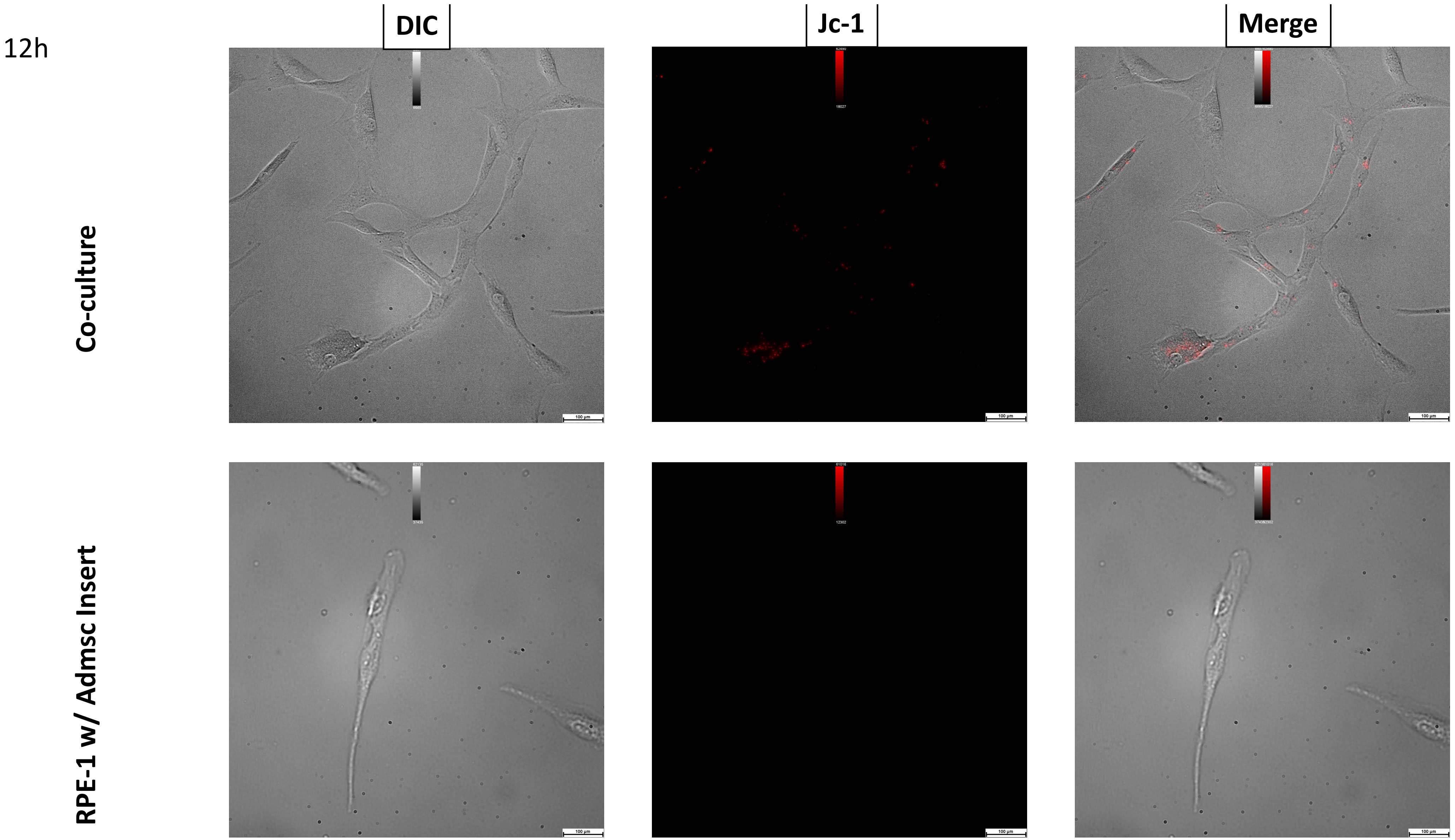
12h
DIC
Jc-1
Merge
Co-culture
RPE-1 w/ Admsc Insert

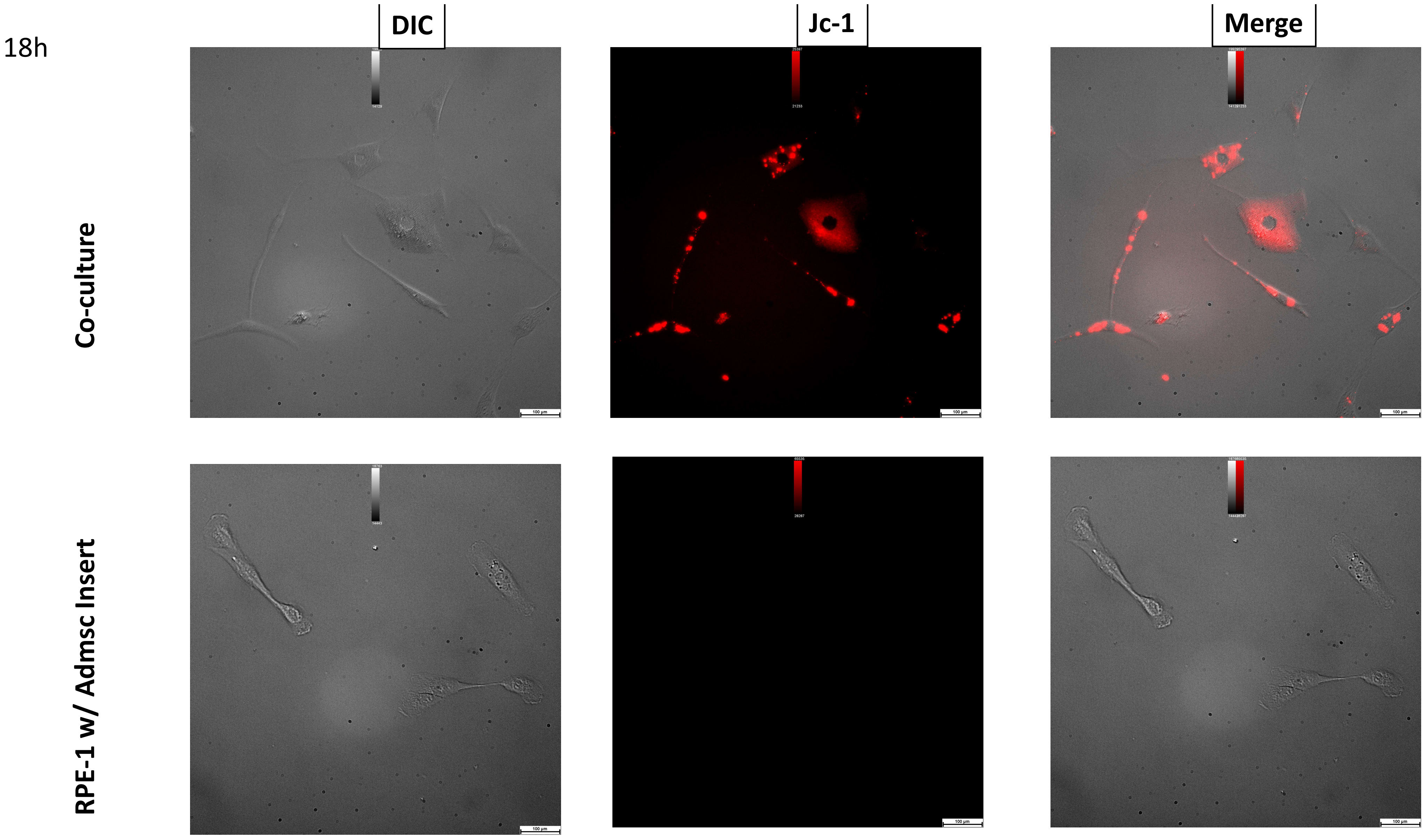
18h
DIC
Jc-1
Merge
Co-culture
RPE-1 w/ Admsc Insert

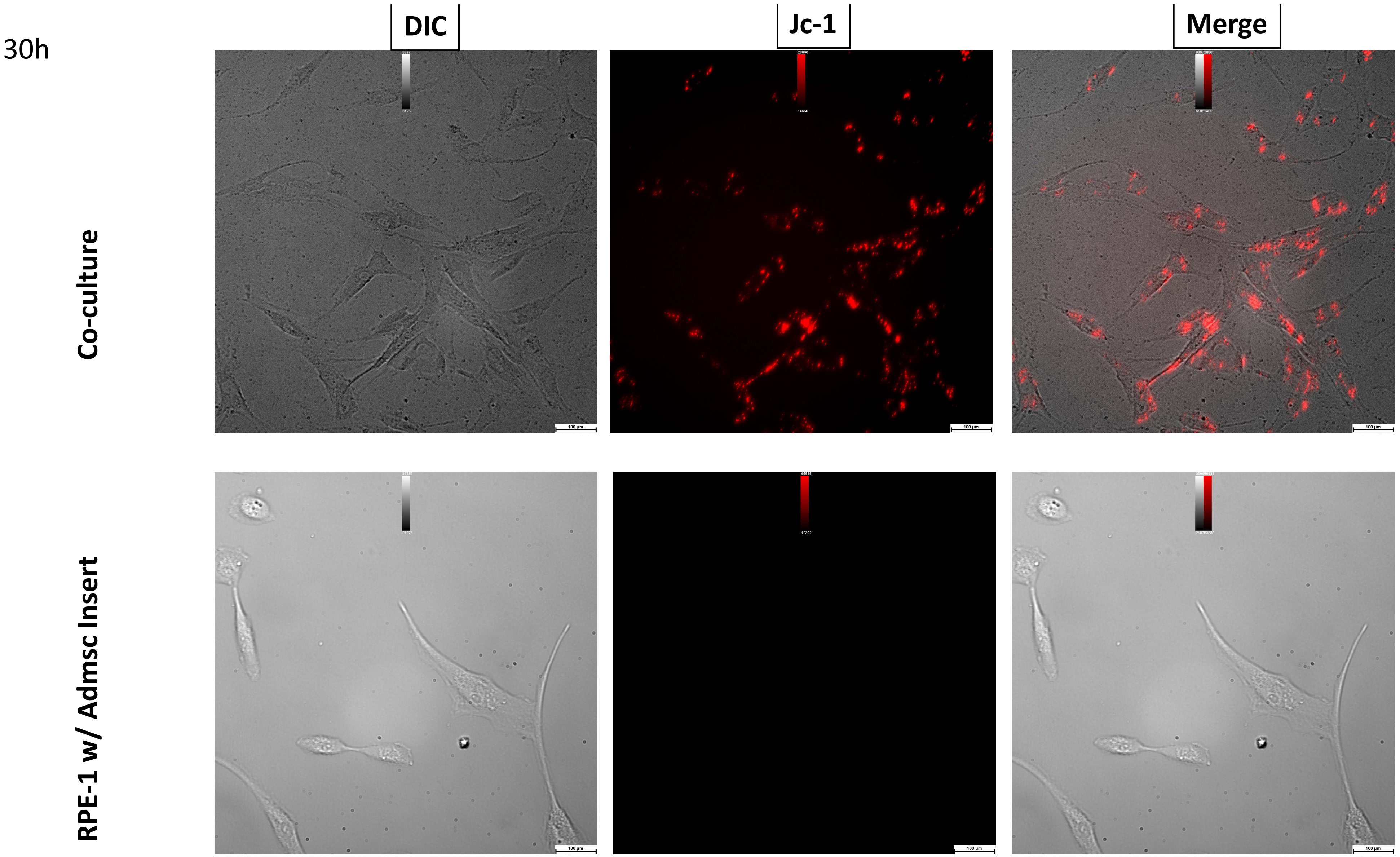
30h
DIC
Jc-1
Merge
Co-culture
RPE-1 w/ Admsc Insert

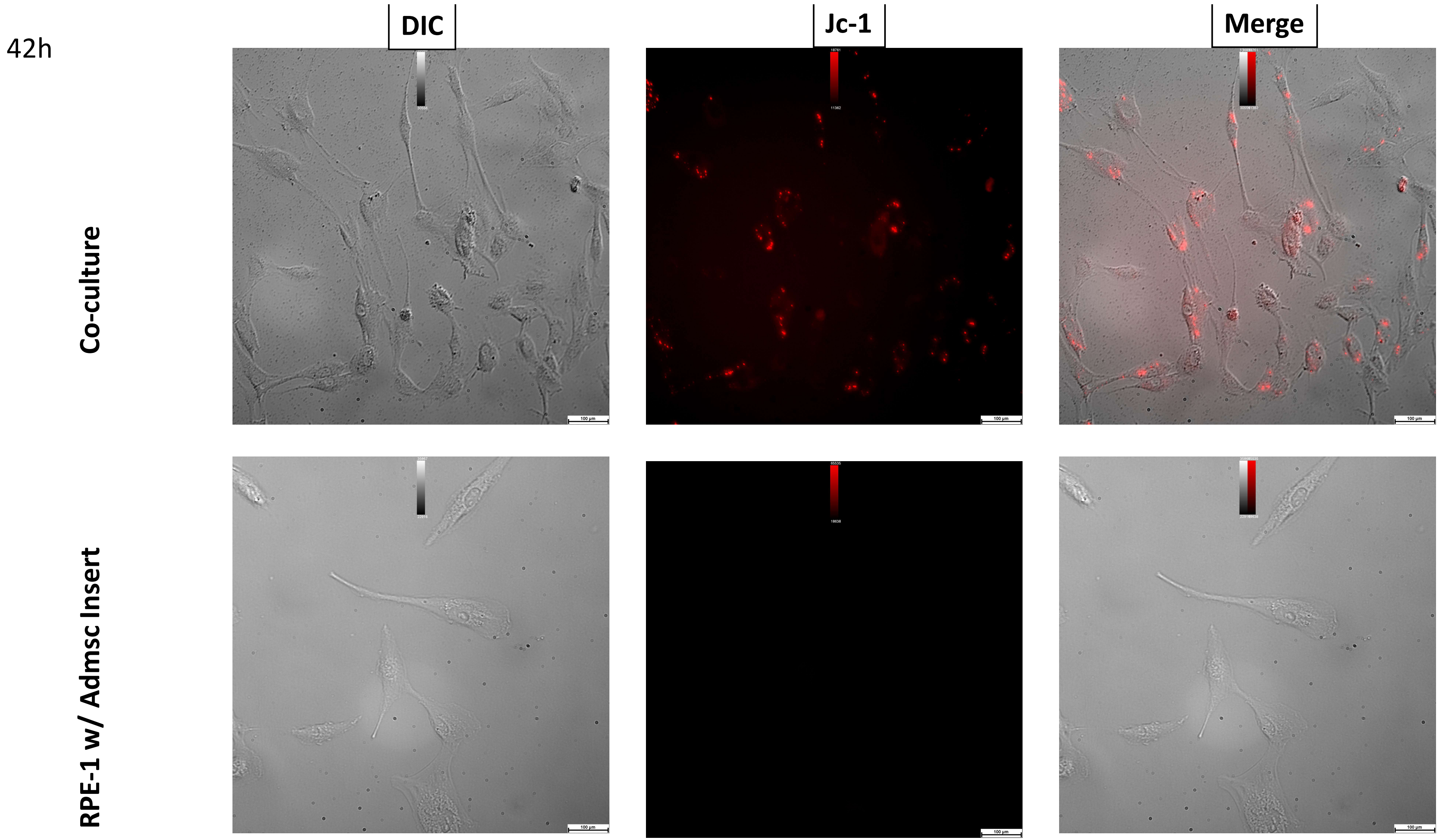
42h
DIC
Jc-1
Merge
Co-culture
RPE-1 w/ Admsc Insert

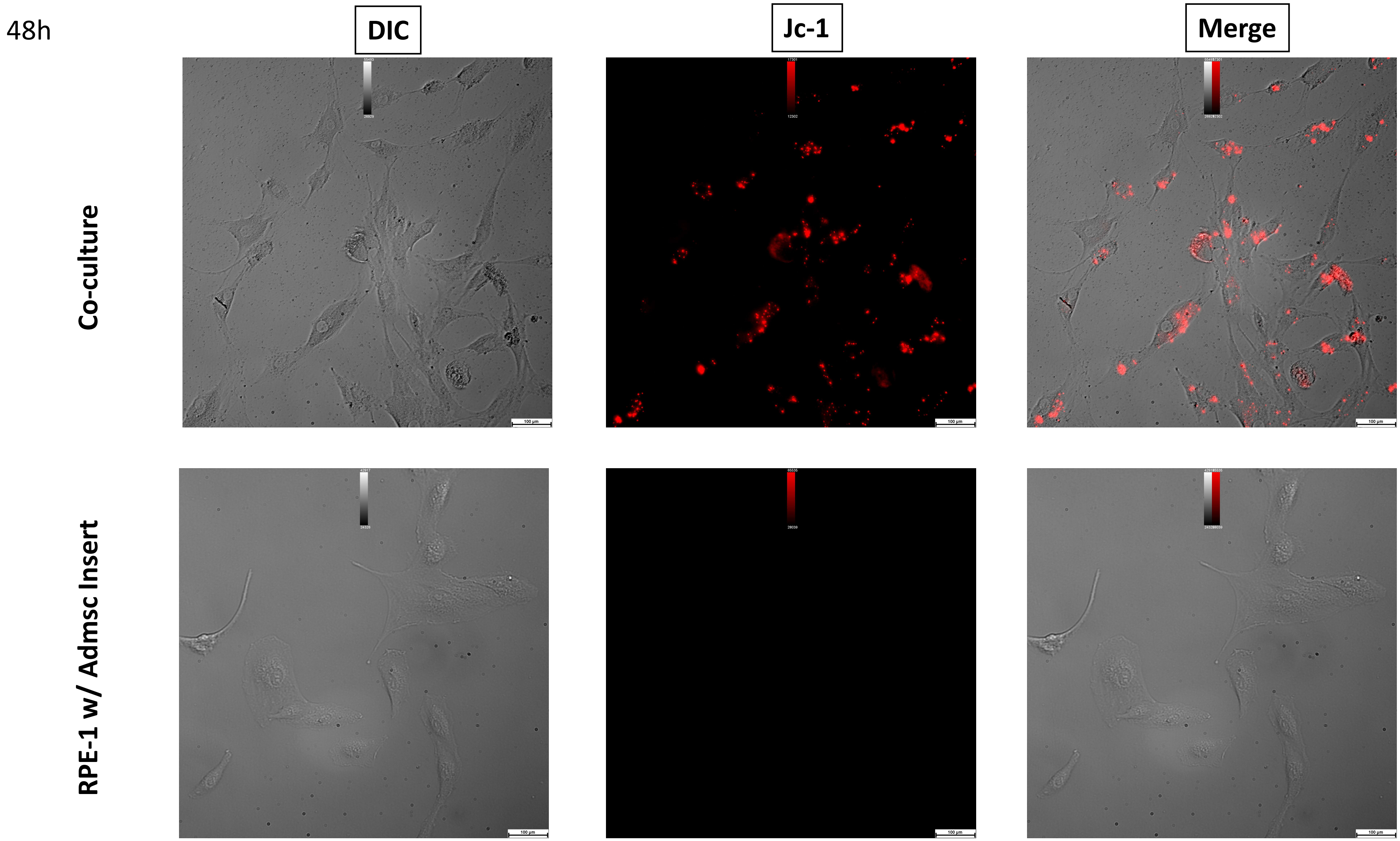
48h
DIC
Jc-1
Merge
Co-culture
RPE-1 w/ Admsc Insert
